# Persistence and Accumulation of Visual Memories for Objects in Scenes in 12-Month-Old Infants

**DOI:** 10.3389/fpsyg.2019.02454

**Published:** 2019-11-06

**Authors:** Sylvia B. Guillory, Zsuzsa Kaldy

**Affiliations:** ^1^Department of Psychiatry, Icahn School of Medicine at Mount Sinai, New York, NY, United States; ^2^Psychology Department, University of Massachusetts Boston, Boston, MA, United States

**Keywords:** visual memory, infants, encoding, persistence, accumulation, interruption, objects, scenes

## Abstract

Visual memory for objects has been studied extensively in infants over the past 20 years, however, little is known about how they are formed when objects are embedded in naturalistic scenes. In adults, memory for objects in a scene show information *accumulation* over time as well as *persistence* despite interruptions (Melcher, 2001, 2006). In the present study, eye-tracking was used to investigate these two processes in 12-month-old infants (*N* = 19) measuring: (1) whether longer encoding time can improve memory performance (accumulation), and (2) whether multiple shorter exposures to a scene are equivalent to a single exposure of the same total duration (persistence). A control group of adults was also tested in a closely matched paradigm (*N* = 23). We found that increasing exposure time led to gains in memory performance in both groups. Infants were found to be successful in remembering objects with continuous exposures to a scene, but unlike adults, were not able to perform better than chance when interrupted. However, infants’ scan patterns showed evidence of memory as they continued the exploration of the scene in a strategic way following the interruption. Our findings provide insight into how infants are able to build representations of their visual environment by accumulating information about objects embedded in scenes.

Natural scenes are semantically coherent images of a real-world environment comprising of background elements (typically larger scale surfaces, such as ground, walls, and floors) and multiple distinct objects (smaller scale entities, such as plants, cars, and chairs) (Henderson and Hollingworth, 1998). Semantic cohesion and regularities aid visual memory performance (Hollingworth and Henderson, 2000; Brady et al., 2009a) and provide contextual cues (Torralba et al., 2006). Visual memory is necessary to accumulate information obtained from the different fixations as the eyes scan the environment (Melcher, 2001; Hollingworth, 2004). This process requires building a complex representation that contains objects that are bound to locations in the scene’s spatial layout and stored in memory (Hollingworth, 2007). The current study investigated how such representations of the visual environment are constructed and maintained in infants.

A fundamental characteristic of visual working memory (VWM) is its limited capacity. Luck and Vogel (1997) found that for adults, VWM stores approximately four units of information. Using a change detection task, adult participants were shown a set of simple objects, such as colored squares; then after a brief delay, the test display was presented where one of the squares may have changed in color. Participants were instructed to indicate whether the two displays were the same or different. These results and similar studies suggest an upper limit to the number of items that can be individuated and maintained (Cowan, 2001, 2010; Scholl and Xu, 2001; Vogel et al., 2001; Alvarez and Cavanagh, 2004; Awh et al., 2007). How much information can be actively stored in VWM has significant consequences on learning and other adaptive functioning (Fukuda et al., 2010).

In development, an emerging picture reveals a gradual increase in VWM capacity over the first year (Rose et al., 2001; Kaldy and Leslie, 2003, 2005; Oakes et al., 2006, 2017; Kibbe and Leslie, 2011; Kwon et al., 2014; Kaldy et al., 2016) that continues to develop into childhood (Simmering, 2012; Guillory et al., 2018). Using a version of the change detection task with three objects, Oakes et al. (2006) found that 8-month-old infants succeeded at binding objects to their locations. Kaldy and Leslie (2003, 2005) reported that 9-month-old, but not 6-month-old, infants looked longer when two objects unexpectedly switched locations. VWM capacity has also been studied in older infants using a manual search paradigm. In these studies, objects are placed into an opaque box and infants are later given the opportunity to search the box and retrieve the hidden objects. Results show that 10- and 12-month-olds are successful in remembering three objects but failed at the task when the quantity was greater than three (Feigenson and Carey, 2003, 2005). Interestingly, 14-month-old infants can use high-level strategies such as chunking to remember more items (Feigenson and Halberda, 2004; Kibbe and Feigenson, 2016). Together, these findings demonstrate that the amount of information and the relationships between objects that can be maintained in VWM develops significantly between 6 and 14 months. However, many important questions remain open about how these processes operate in infants.

The influence of context on object perception has only recently been explored in infants. Examining eye gaze patterns of natural and artificial scenes, object-context congruency, and relational memory has revealed that 4-month-old infants fixate more on objects than the background in natural scenes (Bornstein et al., 2011a), and on objects that are congruent than incongruent with the scene context (Bornstein et al., 2011b). Nine-month-old infants can learn arbitrary face-scene associations (Richmond and Nelson, 2009), and by 12 months, some aspects of their scene scanning and fixation patterns are similar to adults’ such as an early exploratory period with short fixations (Pannasch et al., 2008; Helo et al., 2016), and they also showing differences in the degree that saliency influenced eye movements (Helo et al., 2014).

Here we investigated how infants accumulate information to build a rich representation of objects embedded in scenes over time and interruptions, where interruptions consisted of an exposure to an intervening scene between repeat exposures of the same scene. Research in adults found that memory capacity estimates increased with exposure time when real-world objects were embedded in naturalistic scenes (Melcher, 2001, 2006; Brady et al., 2016). This is contrary to research using monochromatic, geometric objects without a rich background that report a plateau in performance after a certain exposure period (Luck and Vogel, 1997). The semantic richness of real-world stimuli and their familiarity was speculated to enhance memory performance (Brady et al., 2011). Increasing exposure times with these stimuli allowed adults to construct a more robust memory representation that was less prone to decay over time.

Another factor that can influence the robustness of visual memories is interruption that can disrupt the encoding and consolidation process. In the real world, objects are often occluded for brief periods because of changes in the environment or changes in body positioning. Visual memory is essential in maintaining representations over these periods. However, some studies in adults found that brief interruptions caused by intervening stimuli did not significantly impact memory performance (Melcher, 2001, 2006; Melcher and Kowler, 2001). Surprisingly, adults demonstrated similar memory performance when presented with a continuous presentation of displays with objects in a scene compared to when the same displays were presented in intervals that added up to the same duration. That is, interruptions (even up to 20–30 s) did not interfere with the gradual accumulation of visual information. Together, these findings demonstrate both a gradual accumulation over time and persistence over brief periods of interference in adults for objects in scenes.

Only a few studies have explored the effects of interruptions in infant VWM encoding so far. Kaldy and Leslie (2005) reported that when 6-month-old infants saw two items hidden sequentially, they could only remember the features of the last-hidden object. A control study demonstrated that this failure was not due to decay over time: 6-month-olds were successful with the same occlusion time but without an interfering event (the hiding of the second object). In 10- to 14-month-olds, the maintenance of a memory trace was found to be dependent on the number of intervening items, and exceeding capacity limits lead to catastrophic forgetting (Feigenson and Carey, 2003, 2005; Cheries et al., 2006). These results indicate that although infants’ memory capacity is increasing during the first year of life, their VWM is more susceptible to interference during maintenance and may be significantly less durable than adults’.

Our goal in the current study was to examine infants’ ability to accumulate visual memories for objects in scenes and test whether those memories can persist over interruptions in order to identify factors contributing to infants’ memory limitations in real-world settings. In adults, Melcher (2001, 2006, 2010) found increased accuracy with longer encoding periods with no decrement in performance when encoding was interrupted. We adapted this paradigm to be suitable for young infants. We manipulated exposure time to measure whether there was evidence of accumulation and introduced interruptions to investigate whether there is persistence of memories over repeated exposures. To evaluate infants’ memory performance, we measured looking times to the changed object (a novelty preference-based process). We also tested a sample of adults to replicate the effects of accumulation and persistence using our stimuli and to serve as a comparison for infants’ performance (with only minor procedural modifications, see section Materials and Methods). We hypothesized that similar to adults, longer encoding times will lead to improvements in infants’ memory performance; however, unlike in adults, infants’ performance will be lower when the same encoding time is broken up into multiple exposures.

## Materials and Methods

In this experiment, infant and adult participants’ visual memory was assessed using a change detection paradigm. Two experimental conditions were contrasted: continuous exposures and repeat exposures (see Table 1). In continuous exposure trials, participants viewed a computer-generated scene with a fixed number of objects and exposure time was varied. This encoding phase was followed by a test display, where we measured whether participants could identify which of the objects had changed. In repeat exposure trials, an intervening scene was presented between the exposures of the scene. The final exposure was followed by a test display, just as in the continuous exposure condition. The two trial types were presented in a mixed block with trials presented in a fixed pseudorandom order. Manipulation of participants’ encoding time allowed us to test memory accumulation and the manipulation in the number of repetitions of exposure the persistence of the memory for the objects in the scene.

**Table 1:** Trial types and durations (in seconds).

Group	Cont. (one exposure)	Two exposures	Four exposures
Adults	1, 2, 4	1 + 1, 2 + 2	1 + 1 + 1 + 1
Infants	3, 6	3 + 3	

### Participants

Twenty-three adults (female: 15, mean age = 24.8 ± 4.8 years) participated in the adult version of the experiment. Adult participants were undergraduate and graduate students from the University of Massachusetts Boston. The participants were 56.5% Caucasian, 8.7% Black/African-American, and 34.8% Asian. The sample size was based on prior studies by Melcher (*N* = 6–21: Melcher, 2001, 2006), and provides sufficient statistical power to detect differences between the conditions.

Nineteen full-term, healthy 12-month-old infants (female: 6, mean age = 12;09 ± 0;25, month; days) participated in the experiment. One infant was excluded from analysis due to fussiness, resulting in a final sample of 18 infants. Of the 75.7% of parents that provided information on racial background, 64.3% identified as Caucasian, 7.1% as Black/African-American, 14.3% as Asian, and the remaining 14.3% as multi-racial. We determined the appropriate sample size based on the results of pilot study with 8- to 16-month-old infants (Guillory et al., 2015), that showed that a minimum sample size of 11 infants was sufficient to detect a difference between conditions with 80% power and an alpha level of 0.05 (G*Power 3.1, see Faul et al., 2007).

All participants had normal or corrected-to-normal vision with no history of colorblindness in the family. Written informed consent was obtained for all participants from the parent/legal guardian and the University of Massachusetts Boston’s Institutional Review Board approved the study protocol.

### Apparatus and Stimuli

**Figure 1:**
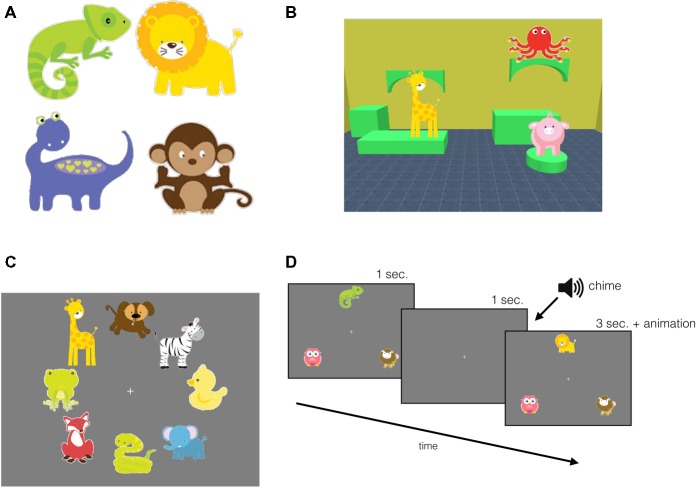
Experimental stimuli and familiarization trials. **(A)** A sample of the objects used in the study. **(B)** An example scene for the *infant version*: scene featuring three objects and the “room” comprised of a unique combination of furniture, wallpaper, and floor tiling. A sample display of **(C)** object familiarization, and **(D)** task familiarization trial sequence, where the top object changed identity after a 1-s delay.

**Figure 2:**
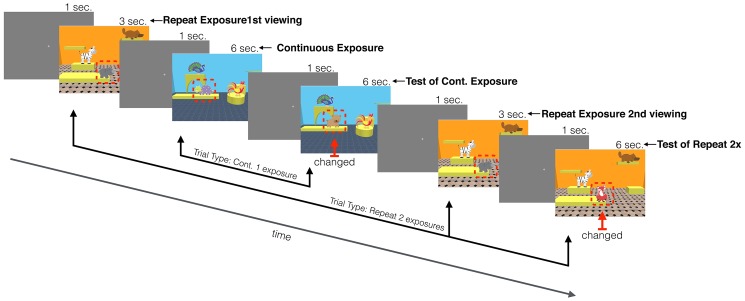
A schematic of the *infant version* of the task, showing a continuous trial interleaved between the repeat exposures (changed objects are shown with a red arrow and outlined with a dashed red line that was not present during actual testing).

A Tobii T120 17-inch eye-tracker (Tobii Technology, Stockholm, Sweden) with a screen resolution of 1,024 pixels × 768 pixels at 32 bits per color and a refresh rate of 60 Hz was used for data collection. Eye gaze coordinates were collected at 60 Hz. The scenes (virtual rooms) were generated using the Sweet Home 3D software application. The rooms consisted of pieces of furniture, wallpaper, and floor tiling. There were 10 possible colors used for both the wallpaper and the furniture, and six possible textures for the floor tiling. By manipulating the color combination of each feature, we generated 60 unique rooms (scenes). Furniture consisted of a collection of highly abstract cylindrical or block shapes, which created flat surface areas for the objects to be placed on, and for a given room consisting of approximately 4–6 surfaces. The objects were selected from a database (Blackleaf Studios, www.mygrafico.com) of colorful cartoon drawings of animals (Figures 1, 2 provide examples of object placement). There were 40 different objects and each object was approximately of equal area, 17,450 pixels^2^ (see examples in Figure 1A). Objects were placed within the scene to avoid any one object obstructing the other.

There were two versions of the task, one designed for infants that contained fewer objects in a trial, longer exposure times, and shorter inter-trial delays, henceforth referred to as the *infant version*, and a task designed for adults that had more objects, shorter exposure times, and longer inter-trial delay periods (*adult version*). Stimulus encoding times of 4 s in duration have demonstrated to be sufficient in achieving above-chance memory performance with 10-month-olds (Kaldy et al., 2016).

The *infant version* of the task contained three objects per scene (Figure 1B) that were placed on three of the 4–6 potential surfaces in the room. Tobii Studio 3.2 software was used to present and collect eye gaze data. Each object was shown once before the test trials during the object familiarization period. For the *adult version*, each room comprised of six objects that were placed on the top surface of the furniture that created the virtual scene and Psyscope XB70 software was used to present the stimuli and record manual responses. Individual differences in capacity limits have found a range of capacity estimates depending on parameters and test procedures; here we used six, as it seemed that that was unlikely to result in ceiling effects (Cowan, 2001; Brady et al., 2016).

### Procedure

Infants viewed the videos while seated on a caregiver’s lap, approximately 60 cm from the screen. The test session started with a standard infant-friendly 5-point calibration. The experiment consisted of three parts: object familiarization, task familiarization, and test trials that were run consecutively without a break, lasting approximately 5 min.

The first phase of the experiment began with object familiarization. To expose the participant to each object once and reduce novelty effects during the task, eight objects were displayed simultaneously on a grey background radially around the central fixation for 10 s (Figure 1C). A blank screen followed each display for 1 s (2 s in the *adult version*) with a fixation cross in the center. There were five object familiarization trials presented, exposing participants to all 40 objects that were used in the test trials. Next, following object familiarization, there were three task familiarization trials designed to expose participants to our change detection task: to familiarize them with the sequence of events and the chime which served as a signal that a test scene was about to appear, followed by the feedback animation. In the task familiarization trials, three objects appeared in a triangular formation with one object above central fixation and two to the bottom right and left of the central fixation on a grey background. The set of three objects were displayed for 1 s after which they disappeared. After 1 s, participants heard a chime intended to serve as a cue that a memory test for the previously presented display would follow. When the objects reappeared, one of the objects was replaced with a novel object. After 3 s, this novel object (target) was animated with an accompanying sound effect, which served as feedback (Figure 1D). These familiarization phases were incorporated in the experiment to diminish novelty effects in a similar fashion that recognition memory studies habituate infants to an image (Fagan, 1972, 1973). In the object familiarization trials, we presented all 40 objects at least once, while minimizing overall session duration. Although infants might not have fixated all of the objects, the size of the attentional window can capture more than just the fixated objects, even in infants (Hernández et al., 2010; Ronconi et al., 2016). The goal of the task familiarization trials was to make learning the event sequence easier for infants: in these trials, the three objects always appeared in the same location to reduce the need to scan the display, and instead of a complex scene context, the objects were presented on a monochromatic background.

These two familiarization phases were followed immediately by the test trials. Each test trial started with a central fixation cross that was presented for 1 s (3 s in the *adult version*). Infants were then presented with a scene that was one of two duration lengths: 3 or 6 s (the *adult version* contained three exposure lengths: 1, 2, and 4 s). A fixation screen followed this scene and then either a test scene (6 s for both *infants* and *adults*) or another scene was presented, depending on trial type: continuous vs. repeat exposures (Figure 2 shows a schematic of a sequence of trials). A continuous exposure trial type consisted of one scene that was immediately followed by the test scene. The repeat exposure trial type consisted of an exposure to an initial scene, followed by an intervening, different scene trial, then a repeat exposure to the initial scene, and finally a test scene. This intervening scene involved different objects and room configuration. In the *infant version* with a maximum of two repeats, the intervening scene was always a continuous trial type, and in the *adult version* consisted of up to four repeats where the intervening scenes were of both continuous and repeat trials intermixed, similar to the procedure used in Melcher (2001, 2006). Which of the three locations had the changed object was randomly selected (the absolute locations were constrained by the room configuration of each scene).

Altogether, there were three different trial conditions in the *infant version* and six trial conditions in the *adult version* (Table 1). Adults were presented with 10 trials of each trial condition for a total of 60 trials. Infants were shown 15 trials: six trials were of the 3-s continuous exposure trial condition, five were the 3-s repeat exposure condition, and four trials were the 6-s continuous exposure condition. Before each memory test display, participants heard a chime. The test display consisted of three objects that appeared in the same locations as the objects shown during the exposure period with the exception that one of the three objects was replaced with a novel one. Adults were presented with test scenes where three of the original six objects were marked by numbers (that is, a partial report test). The test scene was always displayed for 6 s (in both the adult and the infant versions). For each age group (infant and adults), rooms, objects, and object placements were the same across participants.

### Data Analyses

#### Adult Version

Following the procedures of Melcher (2001), adult participants were instructed to select *via* button press, one item out of the three marked objects that changed (selected from the set of six presented in the original scene). Each object in this marked subset was labeled as 1, 2, or 3 (the numbers appeared above the objects) and participants used a Dell keyboard to give their responses. Correct trials consisted of trials where the subject correctly identified the changed object within the 6 s of the test display (before the start of the feedback animation). Trials were categorized as incorrect when the participants selected an object that did not change during the response period (6 s) or responded after the end of this period (during the feedback animation).

#### Infant Version

We calculated a preference measure based on proportional looking: during the test scene, the time spent looking at the target object was divided by the total time spent looking at the three objects. This measure was compared to chance (33%). The default Tobii fixation filter was used for data analysis. Areas of interest were defined as equal-sized rectangular areas surrounding each of the objects (AOI size: 240 pixels × 240 pixels).

### Object and Task Familiarization

One-sample *t*-tests, Bonferroni corrected, were used to analyze the test phase of each of the three task familiarization trials. There were two missing values from two different infants that never looked at the screen during one of the three trials; therefore, instead of a repeated measures ANOVA, one-sample *t*-tests compared the proportion looking time to the target object in each of the three trials to chance performance (33%).

### Memory Accumulation

To analyze whether participants’ memories accumulated over time, performance in the continuous trial types with the different durations were compared to each other. In the *adult version,* this analysis consisted of three different durations (1, 2, and 4 s) and in the *infant version,* two durations (3 and 6 s). Here, we applied a linear regression analysis to determine whether there was a relationship between the duration of exposure and memory performance. In addition, in the *infant version,* we performed one-sample *t*-tests comparing proportion looking results with chance performance (33%) to determine whether infants showed a novelty preference for the new object in the test scene in each trial type.

### Memory Persistence (Resistance to Interruption)

To analyze whether shorter repeated exposures were equivalent to a continuous exposure of the same total duration (e.g, a 2-s exposure repeated twice for a total duration of 4 s results in a similar memory performance in a continuous trial of 4 s), we performed a repeated measure analysis of variance (ANOVA) and paired sample *t*-tests on the proportion looking measure.

Additional exploratory analyses were performed to further examine infants’ performance. We explored the persistence of memory between the two exposures in the repeat exposure trials. We compared proportion looking during the first vs. the second exposure per object and used one-sample *t*-tests to determine whether objects were viewed for similar lengths across exposures.

### Memory Accumulation (Infant vs. Adults)

Lastly, the regression coefficient, *β*, of adults and infants was compared, testing the null hypothesis that the coefficients are the same (*β*_adults_ = *β*_infants_). We achieved this by adding a predictor term to a regression model that reflected the interaction of the two factors [Group (adult/infant) and Encoding time] where the adult sample served as the reference group. The interaction term corresponded to the coefficient difference between groups (*β*_infants_ − *β*_adults_), such that no significant difference indicated no difference in slope.

## Results

### Memory Accumulation—Adults

**Figure 3:**
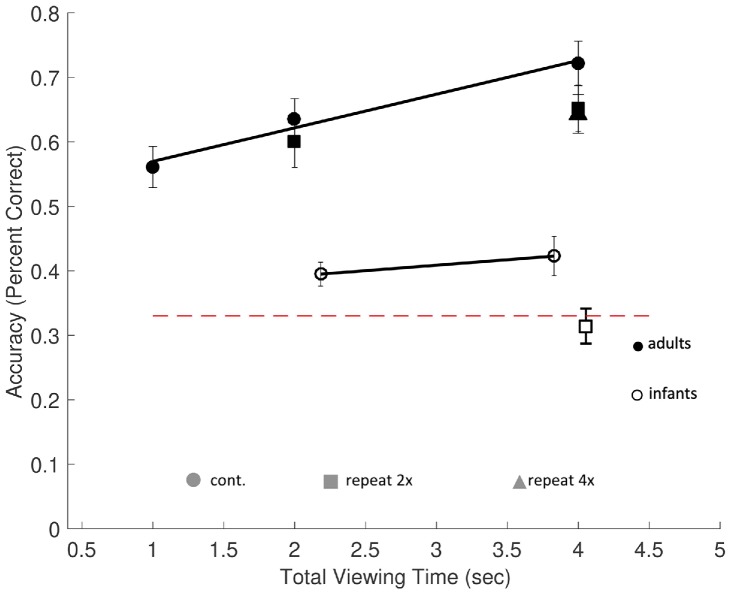
Memory performance in Experiment 1. The rates of encoding time predicting memory performance (percent correct), in adults (filled symbols) and infants (open symbols), with chance performance defined as 33% (dashed red line). Accuracy was based on proportional looking time to the changed object during the test scene in infants and manual responses in adults. Average actual infant looking time on screen is represented on the horizontal axis. Results from continuous exposure trials (circles) and repeat exposure (square and triangle symbols) are shown. Memory accumulation effects among continuous exposure conditions are shown by the solid regression lines. Errors bars are ±1 SEM.

All trials were valid and included in the analysis. To determine whether there was a significant accumulation of information as encoding time increased, a linear regression was applied to predict performance from the total encoding time during the continuous trials (1, 2, and 4 s). Performance significantly increased with increased encoding time [*F*(1,67) = 11.85, *p* = 0.001], with a model fit of *R*^2^ = 0.15 (Figure 3). Participants’ predicted accuracy increased by 3.8% for each second of additional encoding time, *t*(67) = 3.4, *p* = 0.001. These results replicate prior findings in similar tasks that showed that increased encoding time improves recall performance for complex, real-world objects in scenes (Melcher, 2001; Brady et al., 2009b).

### Memory Persistence—Adults

The persistence of the memory representations was tested with a repeated measures ANOVA by comparing the three conditions when the total exposure time was 4 s (4 s continuous, 2 s × 2 s repeat exposures, and 4 s × 1 s repeat exposures). The main effect of condition was not significant, *F*(2,44) = 1.82, *p* = 0.17, $\eta_{p}^{2}$ = 0.076. The same was true when comparing the two conditions where the total exposure time was 2 s (2 s continuous, 2 s × 1 s repeat exposures), *F*(1,22) = 0.67, *p* = 0.42, $\eta_{p}^{2}$ = 0.030. Together, these results suggest that viewing a scene for 4 continuous seconds is equivalent to viewing a scene twice for 2 s, or four times for 1 s, showing an essentially lossless memory representation in adults despite intervening scenes.

### Familiarization—Infants

Object familiarization trials: average looking time to individual objects during these trials was 1.45 ± 0.43 s (means and standard errors are reported from here on). Results for each individual object within our set of 40 objects were within three SDs of this mean.

Task familiarization trials: applying one-sample *t*-test, the proportion looking at the target compared to chance (0.33) during the reappearance of the objects was significantly higher in the first trial [*t*(16) = 4.00, *p* = 0.003]. In the remaining two trials there was significant to a marginally significant difference of below chance looking [second: t(17) = −2.61, *p* = 0.054; third: *t*(16) = −2.82, *p* = 0.036]. Overall proportion looking was comparable across the three familiarization trials [the main effect of Trial was not significant: *F*(1.48,22.18) = 0.614, *p* = 0.50]. It should be noted that (unlike in the test trials), the objects in familiarization trials appeared in the same three canonic (left, right, top center) locations on each trial.

### Test Performance Summary—Infants

Of the 15 test trials presented, on average 3.4 ± 0.7 trials were excluded for each participant for insufficient eye gaze data. Valid trials required that infants looked at the scene during the encoding period (*M* = 2.51 s, SD = 0.09 s during exposure and *M* = 3.72 s, SD = 0.21 s during test). A minimum threshold for a fixation of at least (60 ms) within one of the three objects’ AOIs was used, taking into account the limited temporal resolution of the Tobii T120 (Tatler and Vincent, 2008). Not surprisingly, infants typically did not look at the scenes continuously during the entire exposure period. Their actual average looking times were 2.18 ± 0.08 s in the 3-s continuous exposure condition, a total of 4.05 ± 0.17 s in the 3-s repeat exposure condition, and 3.83 ± 0.21 s in the 6-s continuous exposure condition. In order to facilitate comparisons between infant and adult results, we used these observed looking time values when plotting infants’ results (see Figure 3).

### Memory Accumulation—Infants

The continuous trial types (3 and 6 s) were analyzed to test for memory accumulation. One-sample *t*-tests were used to test whether infants’ proportion of looking time at the changed object was different from chance. Infants performed significantly better than chance (33%) in both the 3-s continuous exposure [*M* = 0.39 ± 0.02, *t*(17) = 3.49, *p* = 0.008, *d* = 0.82] and the 6-s continuous exposure condition [*M* = 0.42 ± 0.03, *t*(17) = 3.04, *p* = 0.02, *d* = 0.72]. However, in the 3-s repeat condition, performance was not significantly different from chance, [*M* = 0.31 ± 0.03, *t*(17) = −0.58, *p* = 1.00, *d* = −0.12].

In repeated exposure trials, for data to be included in these analyses, infants had to look at the scene during both exposures. Therefore, repeated exposure trials were less likely to meet our inclusion threshold than continuous exposure trials, and our final data set contained fewer valid trials in this condition (*M* = 3.22 ± SE = 0.24 vs. *M* = 3.39 ± SE = 0.16 in the 6-s and *M* = 5.00 ± SE = 0.34 in the 3-s continuous exposure conditions). We investigated whether a lower number of valid trials could have led to the higher variance found in the repeat exposure trials. Analyzing the proportion of valid trials for each condition in a repeated measures ANOVA, we found a significant main effect of trial type, *F*(2,34) = 21.24, *p* < 0.001, $\eta_{p}^{2}$ = 0.56. *Post hoc* tests revealed that infants had a higher proportion of valid trials in the 3-s (proportion: *M* = 0.83 ± 0.06) and the 6-s (*M* = 0.85 ± 0.04) continuous conditions compared to the 2 s × 3 s repeat exposure (*M* = 0.64 ± 0.05) condition (Bonferroni-corrected, *p* < 0.001) contributing to the higher variability of the results in this condition.

In an exploratory analysis, we relaxed our exclusion criteria and included those repeat exposure trials where infants only looked at the scene during the second exposure. When analyzing this expanded data set, there were no significant differences between conditions in the number of valid trials, *F*(1.4,23.77) = 0.24 *p* = 0.71, $\eta_{p}^{2}$ = 0.031. However, a one-sample *t*-test showed that despite a small increase in infants’ overall performance (*M* = 0.33 ± 0.03), the overall pattern of results did not change, and it was still not significantly better than chance [*t*(17) = −0.06, *p* = n.s.]. Surprisingly, infants’ performance was still lower in this expanded data set than in the 3-s continuous condition (0.33 vs. 0.39). Further studies are needed to clarify the source of this difference.

### Memory Persistence—Infants

To examine the persistence of memory representations in infants, we performed a paired sample *t*-test comparing performance in the two conditions when the total exposure time was equal, 6 s (6-s continuous vs. 2-s × 3-s repeat exposures). We found that memory performance significantly differed in the two conditions, *t*(17) = 2.66, *p* = 0.017, *d* = 0.63. Thus, we found that viewing a scene twice for 3 s was not equivalent to viewing a scene once for 6 s for infants.

Infants, unlike adults, cannot be instructed to use all of the available exposure time to encode the objects in the scenes; therefore, we analyzed the effects of infants’ actual encoding times (their looking times during exposures) on memory performance. Results from the proportion looking during continuous exposure trials were subjected to a linear regression to test for effects of accumulation using average looking time for each subject in each condition (continuous trial types). Looking time during exposure was a marginally significant predictor of accuracy, *F*(1,34) = 3.80, *p* = 0.059, with an overall model fit of *R*^2^ = 0.101. Infants’ predicted accuracy increased by 3.2% for each second of additional encoding time. That is, longer encoding times lead to a significant increase in infants’ memory performance.

**Figure 4:**
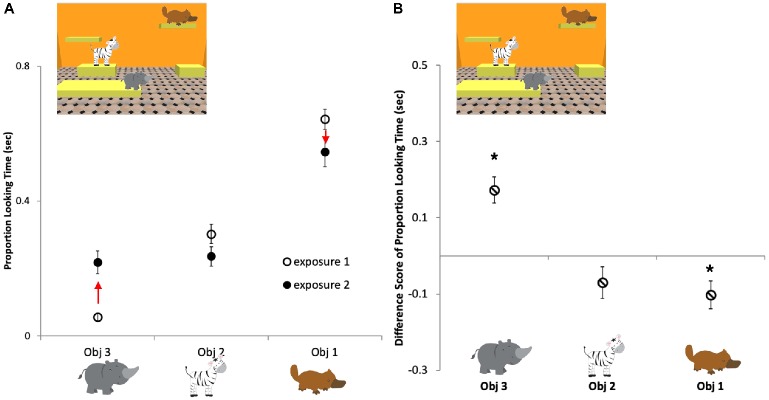
Infants’ average proportion of looking time for each object in the repeat exposure trials. **(A)** First exposure, open circles; second exposure, filled circles; Obj 1, the object that was looked at the longest during Exposure 1; Obj 2, the object that had an intermediate position in looking times during Exposure 1; Obj 3, the object that was looked at for the least amount of time during Exposure 1. **(B)** Average looking time differences between Exposure 1 and Exposure 2 for each of the three types of objects. Infants looked longer at objects in Exposure 2 that were looked at the least in Exposure 1. Conversely, infants looked less at objects in Exposure 2 that were looked at the most in Exposure 1. Asterisks indicate significant differences from zero analyzed by one-sample *t*-tests. Errors bars are ±1 SEM.

To further investigate whether there was any evidence for persistence in infants’ memory over interruptions, in an exploratory analysis, we compared looking times to individual objects during the two exposures in the repeat exposure trials. The proportion of looking time at each of the three objects was calculated for the two exposures separately (Figure 4A) and then the proportion looking during Exposure 1 was subtracted from Exposure 2 to measure the change in the proportion looking at each object (Figure 4B). Using one-sample Bonferroni-corrected *t*-tests, we compared each object’s proportion change value to zero, where zero represents that there was no change in looking time at the object between the first exposure (Exposure 1) of a repeat trial and the second exposure (Exposure 2) of the repeat trial. We found that objects that were looked at the longest initially in Exposure 1 were looked at for a smaller proportion of time during Exposure 2 [−0.10 ± 0.04 s, *t*(16) = −2.81, *p* = 0.04, *d* = −0.68], objects scanned the least during Exposure 1 were looked at longer in Exposure 2 [0.16 ± 0.03 s, *t*(17) = 4.85, *p* < 0.001, *d* = −1.41], while objects that were intermediately attended to, according to this measure during Exposure 1, were looked at approximately the same amount of time in Exposure 2 [−0.07 ± 0.04 s, *t*(16) = −1.69, *p* = 0.32, *d* = −0.41]. These results indicate that infants had some recollection of the objects in the scene when they saw them for the second time and continued to explore them in a strategic way during the second exposure.

### Memory Accumulation—Adults vs. Infants

Lastly, we tested whether infants have a slower rate of encoding in comparison to adults, comparing the regression coefficient (*β*) of adults to infants. Interestingly, the accumulation rates of adults and infants did not differ significantly from one another (*β*_infants − adults_ = −0.021, *p* = 0.43, Figure 3, solid black lines).

## Discussion

The present study aimed to assess the mechanisms used to remember multiple objects in a quasi-naturalistic scene in 12-month-old infants. In particular, our goal was to measure two specific processes of visual memory: accumulation and persistence of visual information (Melcher, 2001; Brady et al., 2009b). We examined this question using a change detection task, contrasting continuous encoding periods with varying length and repeat exposures. While Melcher (2001) used verbal recall to assess memory performance in adults, in our version of the paradigm, we measured recognition memory to make the task appropriate for infants. (This modification did not affect the main pattern of results in adults, which replicated those found by Melcher).

In infants, eye-tracking was used to contrast looking time differences between changed and unchanged stimuli. We found that infants performed significantly better than chance in the continuous exposure conditions of both 3 and 6 s. Our findings are consistent with previous studies showing that 12-month-olds can succeed at VWM tasks involving three objects in a Violation-of-Expectation task using real-world, 3D objects (Kibbe and Leslie, 2013) and that 10-month-olds prefer a changing stream with set-size 3 in a change detection task with a 250-ms delay (Ross-Sheehy et al., 2003).

We replicated previous findings of linear accumulation of visual information over exposure time in adults (Melcher, 2001, 2006). We used two approaches to assess accumulation in infants. First, we looked at percent correct responses in demonstrating a novelty preference for the changed item and found significant increases in performance when exposure time was increased. While our sample consisted of 12-month-olds, it is notable that another study that manipulated exposure times in younger infants found contrasting results. Kwon et al. (2014) found that doubling the exposure time from 500 to 1,000 ms did not have a significant impact on 6-month-olds’ memory performance in a change detection task. Our study design differed from theirs in several ways: we used longer exposures, longer delays between exposure and test, the objects were embedded in scenes, and the infants we tested were older. Taken together, all these factors could have impacted why the infants in our study were able to construct a more durable memory representation with increased exposure.

Adults showed persistence in their memory representations in our study, demonstrating that interruptions did not significantly disrupt their encoding processes (replicating the findings of Melcher, 2001, 2006). We examined memory persistence (resistance to interference caused by intervening stimuli) in infants in two ways. First, we compared accuracy in the continuous (6 s) and repeat exposure (2 s × 3 s) conditions and we found significant differences between them. Infants’ performance in the repeat exposure condition was not significantly better than chance. These results are puzzling as infants were successful in the 3-s continuous exposure condition. In repeat exposure trials, even if infants forgot the objects that they have seen in the first exposure, the second exposure should have resulted in an above-chance outcome. Thus, we conducted an exploratory analysis comparing scanning patterns between the two exposures. This exploratory analysis showed that infants retained some memories between exposures, as they systematically continued their inspection of the objects that they did not look at first. These results are consistent with adult studies that demonstrate memory-guided attention whereby memories influence eye movements during visual exploration (Brockmole and Henderson, 2005; Ryals et al., 2015; Hutchinson et al., 2016).

Previous infant studies that have probed memory performance using a natural scene context have found, that like in adults, regularities that characterize natural scenes influence memory. Duh and Wang (2014) tested 15-month-old infants with objects placed in different natural scenes that were either congruent with the scene gist (fire hydrant in the grass) or not (yellow bottle in the grass). They found that infants often missed salient changes that preserved the overall scene gist, but when the scene gist was disrupted by a change in a non-salient object, infants were able to detect the change. Similarly, 24-month-old toddlers were shown to look longer at objects that were highly salient regardless of semantic consistency, that is, for both congruent and incongruent settings (Helo et al., 2017). In our study, all objects were equally congruent (or incongruent) with the scene gist, and similar to the toddlers in Helo et al. (2017), infants were successful at detecting an object change, indicating that infants were storing information about individual objects in the scene. While there is a lot known about how infants remember object/location pairings in paradigms when the objects are well-segmented and presented without a context, considerably less is known about context-dependent memory through which individual elements are integrated within a scene. Oakes et al. (2011) found that in the presence of spatial reference points (adding a grid around the to-be-remembered items), 6-month-olds’ performance improved when just one object needed to be remembered, but not when the set size increased. Understanding failures and successes on these tasks requires a better understanding of infants’ abilities to build robust associations about objects and their context.

The conclusions we can draw from our results have some limitations. These results likely underestimate infants’ performance, as a very small portion of the images was reused (presented in two different test trials) in the infant study, conceivably leading to a certain amount of proactive interference. Proactive interference arises when previously encoded information interferes with the current contents of working memory (Crowder, 1976), and it has been shown to affect VWM in adults (Makovski and Jiang, 2008). It is also conceivable that infants may show a mixture of preferences for familiar vs. novel objects (Aslin, 2007; Sivakumaran et al., 2018) in this particular paradigm.

In summary, the goal of the current study was to characterize the early development of two specific processes of visual memory for objects embedded in scenes. One of the main objectives was to test whether visual information accumulates over time in young infants. We established that infants performed significantly better than chance in detecting a change in one of three objects and we found memory benefits of increased encoding time on the target object. Our second objective was to investigate whether infants show persistence in information encoding over interruptions, and we found that while infants recognized the objects from previously shown scenes, this did not lead to better recognition performance at this age. Our results open up the field for future developmental work aimed at characterizing the processes underlying the buildup of visual memory representations of objects in scenes.

## Ethics Statement

This study was carried out in accordance with the recommendations of The University of Massachusetts Boston’s Institutional Review Board with written informed consent from all subjects. All subjects gave written informed consent in accordance with the Declaration of Helsinki. The protocol was approved by the University of Massachusetts Boston’s IRB. The legal guardian signed consent to participate in the research study after an experimenter reviewed the consent form and answered any questions that the guardian might have.

## Author Contributions

SG was involved in the study design, data collection, analysis, and writeup. ZK supervised SG and was involved in study conception, design, analysis, and writeup.

### Conflict of Interest

The authors declare that the research was conducted in the absence of any commercial or financial relationships that could be construed as a potential conflict of interest.
